# Targeted next generation sequencing identified novel *loss‐of‐function* mutations in *MERTK* gene in Chinese patients with retinitis pigmentosa

**DOI:** 10.1002/mgg3.577

**Published:** 2019-02-20

**Authors:** Song Liu, Jian Gang Bi, Yunlong Hu, Donge Tang, Bo Li, Peng Zhu, Wujian Peng, Dong Du, Huiyan He, Jun Zeng, Yong Dai

**Affiliations:** ^1^ Clinical Medical Research Center, The Second Clinical Medical College of Jinan University (Shenzhen People's Hospital) Shenzhen Guangdong R.P. China; ^2^ Department of Hepatobiliary Surgery The Second Medical College of Jinan University (Shenzhen People's Hospital) Shenzhen China; ^3^ Department of Pathogen Biology Shenzhen University School of Medicine Shenzhen R.P. China; ^4^ The Third People's Hospital of Shenzhen, Clinical Medical Research Center Shenzhen Guangdong R.P. China

**Keywords:** homozygous, nonsense mutation, novel mutation, retinitis pigmentosa, targeted next generation sequencing

## Abstract

**Background:**

Retinitis pigmentosa (RP) is one of the major types of hereditary retinal dystrophies with extreme genotypic heterogeneity. To date, more than 80 genes have been identified to be associated with RP in human.

**Method:**

Here, we presented a clinical genetic study of three Chinese man manifested with night vision blindness and complete loss of midperipheral visual field. All of these three probands have been identified with loss of both central vision and far peripheral visual field. Gradual loss of rod cells followed by subsequent loss of cone cells have been identified in these probands. Targeted next generation sequencing and Sanger sequencing have been performed to understand the pathogenic variants underlying the disease phenotype in these three unrelated Chinese probands.

**Results:**

Targeted next generation sequencing and Sanger sequencing identified three homozygous novel mutations (c.1880C>A; c.1459_1460delGA, and c.392G>A) in the *MERTK* gene in these three unrelated Chinese proband. In the first proband, the identified mutation (c.1880C>A) leads to the formation of a premature stop codon followed by the formation of a truncated mer‐tyrosine kinase (MERTK) protein (p.Ser627*) product which predicted to be disease causing. In the second proband, the identified deletion (c.1459_1460delGA) leads to the formation of a frameshift which also finally results in the formation of a truncated MERTK protein (p.Asp487Leufs*57) product which also predicted to be disease causing. In the third proband, the identified mutation (c.392G>A) leads to the formation of a premature stop codon followed by the formation of a truncated MERTK protein (p.Trp131*) product which also predicted to be disease causing. Hence, these three mutations are *loss‐of‐function* mutations. These three mutations were absent in unaffected family members and in 100 normal healthy controls.

**Conclusion:**

Our present study also demonstrates the significance of targeted next generation sequencing in determining the genetic basis of RP.

## 1 INTRODUCTION

Retinitis pigmentosa (RP) is one of the major groups of hereditary retinal dystrophies with extreme genetic heterogeneity. Retinitis pigmentosa affects majorly rod cells and it is characterized by gradual and progressive night blindness which finally results in eventual blindness (Busskamp et al., 2010; Hartong, Berson, & Dryja, 2006). Approximately, more than 80 genes have been identified in patients with nonsyndromic RP cases. Among all the nonsyndromic RP cases, 20%–25% are inherited in an autosomal dominant manner, 15%–20% are with autosomal recessive inheritance, and remaining 10%–15% RP cases are with X‐linked inheritance. The rest of the RP cases are sporadic (Fernandez‐San Jose et al., 2005). In addition, molecular genetic analysis has been performed for only 60% of RP cases but 40% of the RP patients lack molecular diagnosis (Daiger, Sullivan, & Bowne, 2013; Wang et al., 2016). Here, we investigated three unrelated Chinese proband, clinically diagnosed with RP. Targeted next generation sequencing identified three candidate novel mutations which helps us to understand the pathology of RP, potential carriers, inheritance pattern, which together finally unveil the novel therapeutic targets.

Germline mutations in *MERTK* gene cause RP [MIM# 613862]. Mer‐tyrosine kinase (MERTK) is a receptor tyrosine kinase that belongs to Tyro3/Axl/Mer family of tyrosine kinases (Boye, Boye, Lewin, & Hauswirth, 2013). Tyro3/Axl/Mer family of tyrosine kinases shares a conserved sequence within the kinase domain and in the adhesion molecule‐like extracellular domain (Tsou et al., 2014). Mer‐tyrosine kinase protein plays a key role in cell proliferation, adhesion, migration, regulation of inflammatory processes, and blood clotting (Zagorska, Traves, Lew, Dransfield, & Lemke, 2014). Mer‐tyrosine kinase protein has a significant role in recycling the outer segment of the photoreceptor cells in the retinal pigment epithelium (RPE). Continuous renewal of the light‐sensitive disks of the outer segment of the rod photoreceptor cells are very important for bearing regular light stress. Phagocytosis of these shaded light‐sensitive disks of the outer segment of the rod photoreceptor cells has been done by RPE (Linger, Keating, Earp, & Graham, 2008). Mer‐tyrosine kinase protein plays a significant role in phagocytosis of the outer segments of the light‐sensitive disks (Nandrot et al., 2007).

Here, in order to identify the molecular basis of RP in these three unrelated Chinese proband from three Chinese families, we performed targeted next generation sequencing with a panel of 60 genes, which has been reported to be associated with RP. In this study, we found three novel homozygous *loss‐of‐function* mutations in *MERTK* gene segregating with RP phenotype in these three Chinese probands, with autosomal recessive inheritance.

## 2 MATERIALS AND METHODS

### 2.1 Ethical statement

Probands and their parents have given written informed consent as they are participating in this study. The Ethics Committee of the Clinical Medical Research Center, The Second Clinical Medical College of Jinan University, Shenzhen People's Hospital, reviewed and approved our study protocol in compliance with the Helsinki declaration. Diagnosis of the patients for RP has been done by ophthalmologist.

### 2.2 Patients and pedigree

**Figure 1:**
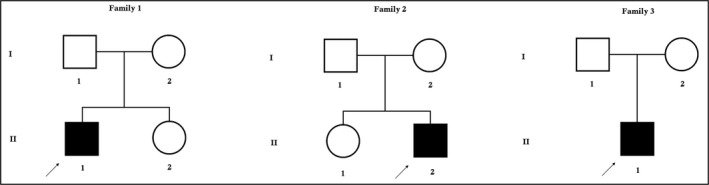
Pedigree of the families. The filled symbol indicates the patient, and the empty symbols show the unaffected healthy parents. The arrow points to the proband

All three probands are of Chinese descend with RP diagnosed in the Clinical Medical Research Center, The Second Clinical Medical College of Jinan University (Shenzhen People's Hospital), Shenzhen, were enrolled in our study (Figure 1).

### 2.3 Targeted exome‐based next generation sequencing and variant identification

DNA samples obtained from the proband (II‐1) were sequenced using target exome‐based next generation sequencing. Roche NimbleGen's (Madison, WI) custom Sequence Capture Human Array was used to designed to capture 221,340 kb of targeted sequence, covering 181 exons and flanking sequence (including the 100 bp of introns) of 60 genes (*ABCA4, AIPL1, ARL6, BEST1, C2orf71, CA4, CDHR1, CERKL, CLRN1, CNGA1, CNGB1, CRB1, CRX, CYP4V2, DHDDS, EYS, FAM161A, FLVCR1,,FSCN2, GUCA1B, IDH3B, IMPDH1, IMPG2, KLHL7, LRAT, MAK, MERTK, NR2E3, NRL, OFD1, PDE6A, PDE6B, PDE6G, PRCD, PROM1, PRPF3, PRPF6, PRPF8, PRPF31, PRPH2, RBP3, RDH12, RGR, RHO, RLBP1, ROM1, RP1, RP2,,RP9, RPE65, RPGR, SAG, SEMA4A, SNRNP200, SPATA7, TOPORS, TTC8, TULP1, USH2A, ZNF513*) associated with RP and yielded an average of 6,366,534 reads per sample, with approximately 96.92% mapping to the targeted regions. The average sequencing depth of the target area is 217.08% with 99.73% coverage. The procedure for the preparation of libraries was consistent with standard operating protocols. In each pooling batch, 10–33 samples were sequenced simultaneously on Illumina HiSeq 2,500 Analyzers (Illumina, San Diego, CA) for 90 cycles (specially designed by us for this study). Image analysis, error estimation, and base calling were performed using Illumina Pipeline software (version 1.3.4) to generate raw data. The raw reads were screened to generate—clean reads‖ followed by established filtering criteria. Clean reads with a length of 90 bp were aligned to the reference human genome from the NCBI database (Build 37) using the Burrows Wheeler Aligner (BWA) Multi‐Vision software package with output files in—bam‖ format. The bamdata were used for reads coverage in the target region and sequencing depth computation, SNP and INDEL calling, and CNV detection. First, a novel three‐step computational frame work for copy number variantion (CNV) was applied. Then, single nucleotide polymorphism (SNPs) and insertion and deletion variant (INDELs) were called using SOAPsnp software and Sam tools pileup software, respectively. A SNP or INDEL was to be filtered if it could not follow the criterion: supported by at least 10 reads and >20% of the total reads. The frequency filter was set at 0.05. If a SNP frequency was more than 0.05 in any of the four databases (dbSNP, Hapmap, 1000 Genomes Project, the 124 healthy reference samples sequenced in this study), it would be regarded as a polymorphism, but not a causative mutation.

**Figure 2:**
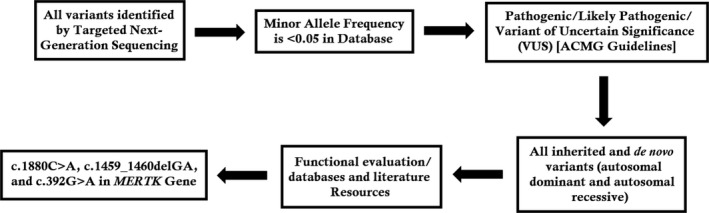
Filtering process for pathogenic mutations in all variations obtained by exome sequencing. Databases used: dbSNP, Hapmap, 1000 Genomes Project

Finally, single nucleotide variantion (SNVs) were retrieved in The Human Gene Mutation Database (http://www.hgmd.cf.ac.uk/ac/index.php) and the Leiden Open Variation Database (http://www.lovd.nl/3.0/home), and then labeled as reported or novel (Figure 2).

### 2.4 Confirmation of the mutation by Sanger sequence

To validate the mutation, Sanger sequencing was performed. Primers flanking the candidate loci were designed based on the reference genomic sequences of Human Genome from GenBank in NCBI and synthesized by Invitrogen, Shanghai, China. PCR amplification was carried out in ABI 9700 Thermal Cycler. PCR products were directly sequenced on ABI PRISM 3730 automated sequencer (Applied Biosystems, Foster City, CA). Sequence data comparisons and analysis were performed by DNASTAR SeqMan (DNASTAR, Madison, WI).

The novel homozygous *loss‐of‐function* mutation identified through targeted next generation sequencing was verified through Sanger sequencing using these three primer pairs: F1 5′‐TTTACCAGTGAGGGACGGGC‐3′, R1 5′‐GTTTGTCTGGCTCCGGTAAGTA‐3′; F2 5′‐GCCACCTATATGGAATAGAC‐3′, R2 5′‐GCGCAGTTTAAGTGGATCCATCG‐3′; F3 5′‐GCAGGCCATTATTGTATAGCGC‐3′, R3 5′‐GCTATAATCTTAGCCAGTGGGAGCATC‐3′. The reference sequence NM_006343 of *MERTK* was used.

## 3 RESULTS

### 3.1 Clinical description

These three probands are 31, 26, and 28 years old man from three unrelated nonconsanguineous Chinese families, respectively (Figure 1). All three probands were manifested with night blindness with consecutive loss of peripheral vision. During ophthalmic examination in all three probands, spicule‐shaped pigmentary deposits have been detected in the fundus with progressive reduction of the visual field in both the eyes. These three probands are only the affected persons in their family. Their parents are phenotypically normal.

All the family members have given their informed consent for participating in this study.

### 3.2 Identification and characterization of candidate mutation

**Figure 3:**
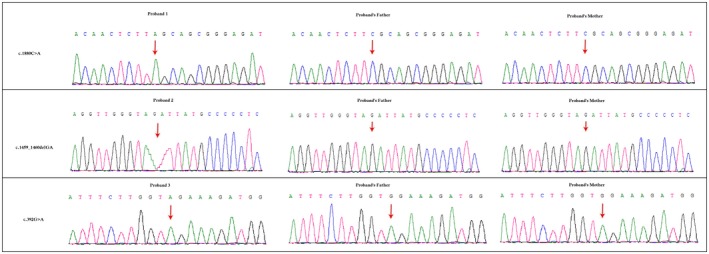
Partial DNA sequences in the *MERTK* by Sanger sequencing of these three probands [NM_006343]. Arrows point to the mutations. These three probands have three homozygous novel mutations; c.1880C>A, c.1459_1460delGA, and c.392G>A

Targeted next generation sequencing and Sanger sequencing identified three homozygous novel mutations (c.1880C>A, c.1459_1460delGA, and c.392G>A) in exon 14, exon 10, and in exon 2 of *MERTK* gene, in these three probands, respectively (Figure 3). These three mutations are predicted to lead to the formation of truncated MERTK protein. In the first proband, the identified mutation (c.1880C>A) leads to the formation of a premature stop codon which in turn predicted to result in premature termination of translation (p.Ser627*), followed by the formation of a truncated MERTK protein. In the second proband, the identified deletion (c.1459_1460delGA) leads to the formation of a frameshift which also finally results in the formation of a truncated MERTK protein (p.Asp487Leufs*57) product which also predicted to be disease causing. In the third proband, the identified mutation (c.392G>A) leads to the formation of a premature stop codon followed by the formation of a truncated MERTK protein (p.Trp131*) product which also predicted to be disease causing. Hence, these three mutations are *loss‐of‐function* mutation. In addition, in silico analysis showed these novel mutations are potential to cause disease (Capriotti, Fariselli, & Casadio, 2005; Schwarz, Cooper, Schuelke, & Seelow, 2014). These three mutations are classified as “*likely pathogenic*” variant based on the variant interpretation guideline of American College of Medical Genetics and Genomics (ACMG) (Richards et al., 2015).

We did not detect this mutation in these three proband's unaffected parents and in 100 normal healthy control individuals of the same ethnic origin, gender, and age range. We did not find these three variants in HGMD, ExAC, dbSNP, and 1000 genomes database.

## 4 DISCUSSION

In our study, we found three homozygous novel *loss‐of‐function* mutations (c.1880C>A, p.Ser627*; c.1459_1460delGA, p.Asp487Leufs*57; c.392G>A, p.Trp131*) [NCBI Reference sequence NM_006343] of the human *MERTK* gene in these three probands. All of these homozygous mutations in *MERTK* gene are predicted to form truncated MERTK protein which is shorter than the wildtype MERTK protein consisting of 999 amino acids. Hence, all these three mutations are *loss‐of‐function* mutations.

Germline mutations in *MERTK* genes are associated with autosomal recessive RP (arRP), which is extremely rare. Clinical manifestations of our patient are similar to all RP patients with mutations *MERTK* gene reported previously.

In our present study, we presented three Chinese proband with RP with an autosomal recessive pattern of inheritance. Additionally, in order to identify the candidate gene mutation, targeted next generation sequencing has been undertaken here for the proband. Our present study also describes the significance of targeted next generation sequencing for clinical application in medical genetics. In contrast with whole genome or whole exome sequencing, targeted next generation sequencing is more advantageous considering the cost and the turn‐around time. Hence, targeted next generation sequencing is an appropriate first‐line tool for genetic screening for the disease with known candidate genes (Weisschuh et al., 2016).

In patients with autosomal recessive disorder, usually harboring homozygous mutation or compound heterozygous mutations, inherited from heterozygous carrier parents. Generally, we found that homozygous mutations or compound heterozygous mutations are inherited in the proband from their parents. In our present study, all these three novel mutations are de novo mutations. It is extremely rare of having homozygous de novo mutation in patients with RP with autosomal recessive mode of inheritance. Hence, this is the novelty of our present study.

In conclusion, here, we report three Chinese patients who presented with RP, with novel mutations in the *MERTK* gene. Our study is significant for genetic screening and clinical diagnosis of patients with RP.

## ETHICS STATEMENT

We obtained written informed consent for genomic analysis of the patient and his family members in accordance with the Declaration of Helsinki. The project was approved by the ethics committee of the Clinical Medical Research Center, the Second Clinical Medical College of Jinan University (Shenzhen People's Hospital), Shenzhen, Guangdong, China and informed consent was obtained from all participants. The proband and his family members provided written informed consent for the publication of the patient's identifiable information.

## CONFLICT OF INTEREST

None declared.
